# School and Bicultural Factors as Mediators between Immigrant Mothers’ Acculturative Stress and Adolescents’ Depression in Korea

**DOI:** 10.3390/children10061010

**Published:** 2023-06-03

**Authors:** Yangmi Lim

**Affiliations:** Department of Home Economics Education, College of Education, Jeonju University, Jeonju 55069, Republic of Korea; ym68@jj.ac.kr; Tel.: +82-63-220-2338

**Keywords:** depression, acculturative stress, school involvement, school adjustment, bicultural acceptance, immigrant mothers, multicultural families, differences by sex

## Abstract

In Korea, marriages between Korean men and foreign women have surged since the late 1990s, resulting in public interest in the psychosocial adjustment of their children. This study examined the mediating effects of immigrant mothers’ school involvement, adolescents’ school adjustment, and bicultural acceptance on the relationship between the mothers’ acculturative stress and adolescents’ depression, as well as whether the structural relationships differed by the sex of adolescents. Data were collected from 1238 dyads of first-grade students (605 boys, 633 girls; age = 12.97 ± 0.35 years) in Korean middle schools and their immigrant mothers (age = 43.52 ± 5.13 years) who participated in the Multicultural Adolescents Panel Study. The structural equation modeling analysis revealed that mothers’ acculturative stress was indirectly and positively related to adolescents’ depression through (1) the serial mediations of mothers’ school involvement and adolescents’ school adjustment and (2) the individual mediation of adolescents’ school adjustment. Furthermore, the multigroup analysis indicated that the relationships between adolescents’ school adjustment and depression and between adolescents’ bicultural acceptance and depression significantly differed between male and female adolescents. The study provides directions for schools and communities to increase immigrant mothers’ school involvement and to facilitate their children’s school adjustment and bicultural acceptance.

## 1. Introduction

Multicultural societies, characterized by people of different races, ethnicities, and nationalities living together in the same community, have become a universal trend. In Korea, since the late 1990s, migrant workers have become more common due to globalization, while marriage immigrants have increased with local governments’ encouragement of cross-border marriages between rural bachelors—who were often marginalized in domestic marriages—and foreign brides from less developed countries [1]. Accordingly, in Korea, with the increasing number of foreigners, the term “multicultural family,” which means a family comprising members of various nationalities and cultures, has emerged [2]. Among multicultural families, the families of foreign workers, international marriages, and North Korean defectors are usually vulnerable groups in most need of social support [3].

In particular, as international marriages have accounted for nearly 10% of all marriages in Korea since 2010, the families of immigrant women have become the main policy targets [4]. The majority of international marriages occur between Korean men and foreign women. As of 2021, there were 168,611 marriage immigrants in Korea, mostly female (136,668; 81.1%), and the major countries of their origin were China (33.5%), Vietnam (27.7%), Japan (10.1%), and the Philippines (8.4%) [5]. Therefore, in this study, the term “multicultural families” is used to refer to families formed through marriage between a Korean husband and a foreign wife.

Meanwhile, along with the continuously growing number of children from multicultural families in schools, there has also been increased interest in the psychosocial health of their children. The number of students from multicultural families in Korean elementary, middle, and high schools in 2021 was 131,522, representing an increase of 76.9% compared with 2015; this is in contrast to the 12.6% drop in the total number of students due to the birth rate decrease during the same period [6]. Children of multicultural and immigrant families have been reported to experience psychosocial maladjustment [7,8,9,10,11]. Immigrant families, where all family members migrate to host countries or only married couples migrate and give birth to children in host countries [7,8], differ from multicultural families, where only mothers migrate to Korea with marriage. However, children from both immigrant and multicultural families are more likely to develop psychosocial maladjustment than children of non-immigrant and non-multicultural families due to their families’ cultural backgrounds.

Children from multicultural families are exposed to developmental and environmental risk factors that can jeopardize their psychosocial health as they enter middle school. During this period, in addition to the transition to a different school environment from elementary school, children generally experience physical and physiological changes with puberty [12]. Furthermore, middle school students experience more pressure to gain high school grades than elementary school students and suffer more social comparisons with their peers [11]. Depression and anxiety are major psychological problems that can occur in adolescence [13]. In particular, as of 2021, 26.8% of adolescents in Korea had experienced depressive symptoms, a 1.6% increase compared with 2020 [14]. In addition, adolescents from multicultural families, unlike those from non-multicultural families, experience identity crises owing to their parents’ different cultural backgrounds. As of 2021, the majority of the adolescents from multicultural families (92.8%) were born in Korea [6]. However, other risk factors for psychosocial maladjustment in multicultural adolescents include communication problems with their immigrant mothers and bullying by peers due to dissimilar skin colors and appearances. Indeed, adolescents from multicultural families experience more mental health problems such as depression than those from non-multicultural families [15]. Therefore, since adolescents from multicultural families are in an environment that makes them vulnerable to depression, it is necessary to systematically identify the factors related to depressive symptoms and design effective interventions.

Parental, school, and acculturation factors are the primary social and environmental factors influencing depression among the adolescents of immigrant and multicultural families [16]. In particular, parental factors such as acculturative stress, parenting behaviors, and a lack of educational support are major environmental factors affecting adolescents’ psychosocial adjustment [16,17]. Acculturation, which occurs following immigration, refers to the psychological–cultural adaptation in which groups or individuals adjust the social and cultural values, beliefs, and behavioral patterns of their countries of origin to those of the host countries [18,19]. It was originally conceptualized as a unidimensional process in which retention of the heritage culture and acquisition of the host culture were cast as opposing ends of a continuum. According to the unidimensional model, as immigrants assimilated the values, practices, and beliefs of the host countries, they discarded those of their countries of origin. However, since the early 1980s, cultural psychologists have recognized that immigrants’ assimilation into the culture of the host countries does not automatically imply rejection of their heritage culture. As such, multidimensional models of acculturation have been postulated by many scholars. One of the most widely studied multidimensional models is Berry’s bidimensional model [19], wherein receiving-culture acquisition and heritage-culture retention are cast as independent dimensions. Within Berry’s model, these two dimensions intersect to create four acculturation types [18]. Integration, also known as biculturalism, describes accepting both the identities of the heritage culture and host culture without resistance. Assimilation refers to having a strong orientation to the host culture but a low maintenance of the heritage culture, whereas separation is characterized by having a strong maintenance of the heritage culture but a low acceptance of the mainstream culture. Finally, marginalization is defined as the low acceptance of both cultures. As immigrants choose any of these four types while meeting new cultures, individual differences in acculturation outcomes are the result of their specific choices [19]. Studies to date have suggested that those with integration acculturation show the most favorable psychosocial outcomes, whereas those with marginalization acculturation exhibit the worst psychosocial outcomes [18,19].

Acculturation can be a stressful experience for a variety of reasons. Acculturative stress refers to the stress that arises while adjusting to another culture’s food, clothing, climate, housing style, and language compared with one’s native country [18]. Immigrant parents may experience language barriers, discrimination, and social isolation while adapting to their host country’s culture and people, thereby suffering stress and inner conflict [17,18]. Especially, mothers in Korean multicultural families move to Korea alone and then start their families; therefore, they may be forced to assimilate and adjust to the Korean culture by their husbands and parents-in-law. Furthermore, they may suffer acculturative stress due to language and cultural differences, nostalgia for their native country, family conflict, xenophobia stemming from anti-immigrant sentiment, and residence status-related anxiety [17]. Immigrant parents’ acculturative stress has been found to directly affect their children’s depressive symptoms as well as indirectly impact them through parenting behaviors [7,8,9,10]. Especially, immigrant parents with a higher degree of acculturative stress are more likely to develop negative parenting practices—such as lack of warmth or support—or provoke parent–child conflicts, leading to school maladjustment in their children [8,9,10]. Moreover, many studies have empirically found that school adjustment is negatively associated with depression among adolescents [20,21].

Additionally, children of multicultural families in Korea must adapt to the cultures of both Korea and their mothers’ home countries [22]. Nho and Hong [23], by considering the characteristics of multicultural families in Korea, proposed the concept of “bicultural acceptance,” defined as one’s acceptance of both the mainstream culture (the Korean culture) and heritage culture (i.e., the culture of the immigrant mother’s native country). The degree of bicultural acceptance in the children of multicultural families is influenced by the mother’s acculturation, parenting behaviors, and the children’s school adjustment [24,25,26], and this has been suggested to affect depression [27].

However, earlier research has its limitations; specifically, previous studies investigating the factors related to depression among adolescents from immigrant and multicultural families have focused on only one or two parent, school, or acculturation factors and rarely examined the comprehensive pathways through which all these factors affect depression among adolescents. Therefore, the present study aims to identify the effect of immigrant mothers’ acculturative stress on depression among first-year middle school adolescents in Korean multicultural families as well as the multiple mediation processes of mothers’ school involvement, adolescents’ school adjustment, and their bicultural acceptance in the relationship. In addition, this study examines whether there are differences in the structural relationships among these variables according to the sex of adolescents.

### 1.1. Theoretical Background: Family Stress Model and Family Systems Theory

The process of mothers’ acculturative stress influencing their children’s depression is explained by the family stress model and family systems theory. The family stress model posits that the stress experienced by parents (e.g., economic and acculturative stress) affects children’s psychosocial health through the parents’ psychological distress and negative parenting behaviors [28]. Several studies have identified the pathways from immigrant parents’ acculturative stress through their depression or dysfunctional parenting behaviors to their children’s psychosocial maladjustment [7,29].

Family systems theory explains the process by which parents’ acculturative stress influences parenting and thus children’s problem behaviors. According to this theory, the family is an open and interdependent system that interacts with its surrounding environment and in which the family members influence each other; thus, any stress experienced by a family member in a specific area of life outside the home affects their role performance in their family as well as the emotions and behaviors of other family members through spillover and crossover processes [30]. A spillover process occurs at the intraindividual level across different life domains and in the same valence (e.g., immigrant parents’ acculturative stress leads to their hostile or neglectful parenting behaviors), whereas a crossover process takes place at the interindividual level such that stress and the resulting negative emotions experienced by an individual in a particular area of life affect the psychological well-being and behaviors of others (e.g., immigrant parents’ acculturative stress causes problem behaviors in their children). In this respect, a crossover occurs in a relationship between two people who form an emotional bond and spend a lot of time together, either by directly transferring one person’s negative emotions or stress to the other through emotional contagion or by indirectly influencing the other’s behavior and emotion via their interaction [30].

### 1.2. Direct and Indirect Pathways from Mothers’ Acculturative Stress to Adolescents’ Depression: Mediating Roles of Mothers’ School Involvement, Adolescents’ School Adjustment, and Adolescents’ Bicultural Acceptance

Mothers’ acculturative stress directly influences adolescents’ depression and indirectly impacts it through mothers’ school involvement, adolescents’ school adjustment, and adolescents’ bicultural acceptance. As noted above, several studies have found a pathway through which the acculturative stress of parents with immigrant backgrounds directly affects their children’s depression [7,17]. Parents’ acculturative stress accompanies their own depressive symptoms and psychological distress, which then cause depressive symptoms in their children through cognitive empathy or emotional contagion [7]. Moreover, if parents experience psychological distress or depression, they cannot appropriately respond to their children’s behaviors and needs, as they are preoccupied with their own emotions and thoughts, ultimately leading to depression in the children [31].

Furthermore, mothers’ school involvement and adolescents’ school adjustment could play serial mediation roles in the relationship between mothers’ acculturative stress and adolescents’ depression. During childhood and adolescence, when school adjustment emerges as a critical developmental task for children and adolescents, parents’ school involvement (e.g., participation in school activities and parent–teacher meetings) has a significant influence on their children’s school adjustment [16,32,33]. In particular, because the school environment and educational system differ from those in the immigrant parent’s native country, the parents’ school involvement has an even greater influence on providing the necessary support for their children’s school adjustment [16,34]. However, immigrant mothers tend to show lower school involvement than non-immigrant mothers [35]. Acculturative stress factors such as language barriers, discrimination, and prejudice against foreigners have been found to hinder immigrant mothers’ school involvement [36]. Furthermore, immigrant mothers’ lower acculturative stress is associated with higher levels of school involvement [9,37]. Such school involvement by parents improves their children’s school adjustment and academic achievement [9,32,38]. In addition, when adolescents’ school adjustment and satisfaction with peers and teachers are low, they show higher levels of depression [20,21]. Accordingly, a pathway from mothers’ acculturative stress, through mothers’ school involvement and adolescents’ school adjustment, to adolescents’ depression is assumed.

In addition, adolescents’ bicultural acceptance mediates the relationship between their school adjustment and depression in the pathway from mothers’ acculturative stress, through mothers’ school involvement and adolescents’ school adjustment, to adolescents’ depression. Several studies show that children and adolescents have increased bicultural acceptance when they are well adjusted to school and form positive relationships with their peers and teachers [24,39]. Moreover, the higher the bicultural acceptance of adolescents from immigrant and multicultural families, the lower are their depression levels [27,40]. Consequently, a pathway from mothers’ acculturative stress, through mothers’ school involvement, adolescents’ school adjustment, and adolescents’ bicultural acceptance, to adolescents’ depression is hypothesized.

Mothers’ school involvement and adolescents’ bicultural acceptance may also play serial mediation roles in the relationship between mothers’ acculturative stress and adolescents’ depression. When immigrant parents show more interest in education and have higher school involvement, adolescents’ bicultural acceptance is higher [25,41,42]. School is a major environment wherein the children of immigrant and multicultural families interact with peers and adults from mainstream cultural backgrounds. When immigrant parents show an interest in their children’s school life and support their school adjustment through higher school involvement, the children have positive attitudes toward the mainstream culture [41]. In addition, when both parents in multicultural families provide sufficient emotional and instrumental support for their children, children have higher levels of bicultural acceptance [42]. Therefore, considering the relationship between bicultural acceptance and depression [27,40], a pathway from mothers’ acculturative stress, through mothers’ school involvement and adolescents’ bicultural acceptance, to adolescents’ depression is postulated.

Furthermore, as explained by the crossover process proposed by Bakker and Demerouti [30], immigrant mothers’ acculturative stress directly exerts a negative effect on adolescents’ school adjustment. Parents’ acculturative stress and the resulting negative emotions are transferred to their children through emotional contagion, which leads to children’s school maladjustment that manifests as externalizing and internalizing problems at school [43]. Many studies have also identified the negative effects of mothers’ acculturative stress on their children’s school adjustment [10,44]. Therefore, as adolescents’ school adjustment is negatively associated with their depression [20,21], a pathway from mothers’ acculturative stress, through adolescents’ school adjustment, to adolescents’ depression is assumed.

Moreover, considering the aforementioned research findings [24,27,39,40], adolescents’ bicultural acceptance is presumed to mediate the relationship between their school adjustment and depression. As such, it can be hypothesized that mothers’ acculturative stress affects adolescents’ depression through the serial mediation processes of their school adjustment and bicultural acceptance.

Finally, mothers’ acculturative stress could affect adolescents’ depression through adolescents’ bicultural acceptance. According to several studies, when immigrant mothers adopt integrated attitudes toward the cultures of their countries of origin and of the host countries or have lower levels of acculturative stress, their children have higher bicultural acceptance and lower acculturative stress [26,45]. This is because when immigrant mothers successfully adjust to the host country’s culture while maintaining their own heritage culture, they become acculturation role models for their children. Furthermore, such immigrant mothers’ integrative acculturation or lower acculturative stress allows them to deliver the values and norms of both the host country’s mainstream culture and their own heritage culture through parenting behaviors and interactions with their children, which helps their children establish a bicultural identity [46]. Therefore, considering the negative relationship between bicultural acceptance and depression in children from immigrant and multicultural families [27,40], a pathway from mothers’ acculturative stress, through adolescents’ bicultural acceptance, to adolescents’ depression is postulated.

### 1.3. Differences in Structural Relationships According to the Sex of Adolescents

The structural relationships among mothers’ acculturative stress, mothers’ school involvement, adolescents’ school adjustment, adolescents’ bicultural acceptance, and adolescents’ depression hypothesized in this study may differ by the sex of adolescents. Parents tend to apply different socialization strategies regarding the expression of psychological distress according to the sex of their children; specifically, when children become stressed and angry, parents encourage passive and inward emotional expression, such as repressed anger, for girls, whereas they allow outward emotional expression, such as obvious expressions of anger, for boys [47]. Consistent with this claim, Güngör and Bornstein’s [48] study investigating the psychosocial maladjustment of adolescents from immigrant families found that male adolescents showed more externalizing problems, whereas female adolescents exhibited more internalizing problems.

Moreover, girls are socialized to be more empathetic to others’ emotions and highly oriented to interpersonal relationships [49]. Therefore, girls show higher levels of depression than boys when their mothers experience stress or depression [50] and are more negatively affected by dysfunctional parenting than boys [51]. However, given that some research has found that sex differences in children are not found in the effects of mothers’ emotional states, such as depression, on their children’s levels of emotional problems [52], further exploration is needed to identify whether the effects of parents’ acculturative stress and parenting behaviors, such as school involvement, on their children’s levels of psychosocial adjustment (i.e., school adjustment and depression) differ by the sex of the children.

Likewise, the school adjustment and bicultural acceptance of adolescents from multicultural families may affect their levels of depression differently depending on their sex. Male adolescents from immigrant families recognize more discrimination experiences in the host countries, have more difficulties adjusting to the host country’s mainstream culture, and exhibit more behavioral problems than female adolescents [16]. Further, in Turjeman et al.’s [53] study of Soviet Union immigrants in Israel, social support and social relationships in school were found to have stronger effects on female adolescents’ mental health, while acculturation difficulties affected male adolescents’ mental health to a larger degree. Notably, boys are more vulnerable to depression when experiencing acculturation difficulties, as they recognize acculturation in the host country as a major task to be accomplished because they are socialized to be more self-reliant and achievement-oriented than girls [53]. Meanwhile, another study found that the effects of the school adjustment and acculturation of immigrant adolescents on their levels of psychosocial adjustment do not differ by sex [16]; thus, ongoing exploration in this regard is warranted.

### 1.4. The Hypotheses and Research Question of this Study

Based on the previous research findings, this study formulates the following hypotheses and research question.

**Hypothesis** **1.** *Immigrant mothers’ acculturative stress directly and positively influences adolescents’ depression.*

**Hypothesis** **2.** *Immigrant mothers’ acculturative stress positively influences adolescents’ depression through the serial mediation processes of mothers’ school involvement and adolescents’ school adjustment.*

**Hypothesis** **3.** *Immigrant mothers’ acculturative stress positively influences adolescents’ depression through the serial mediation processes of mothers’ school involvement, adolescents’ school adjustment, and adolescents’ bicultural acceptance.*

**Hypothesis** **4.** *Immigrant mothers’ acculturative stress positively influences adolescents’ depression through the serial mediation processes of mothers’ school involvement and adolescents’ bicultural acceptance.*

**Hypothesis** **5.** *Immigrant mothers’ acculturative stress positively influences adolescents’ depression through the mediation process of adolescents’ school adjustment.*

**Hypothesis** **6.** *Immigrant mothers’ acculturative stress positively influences adolescents’ depression through the serial mediation processes of adolescents’ school adjustment and bicultural acceptance.*

**Hypothesis** **7.** *Immigrant mothers’ acculturative stress positively influences adolescents’ depression through the mediation process of adolescents’ bicultural acceptance.*

**Research** **Question.** *Do the relationships among mothers’ acculturative stress, mothers’ school involvement, adolescents’ school adjustment, adolescents’ bicultural acceptance, and adolescents’ depression differ by the sex of adolescents?*

**Figure 1:**
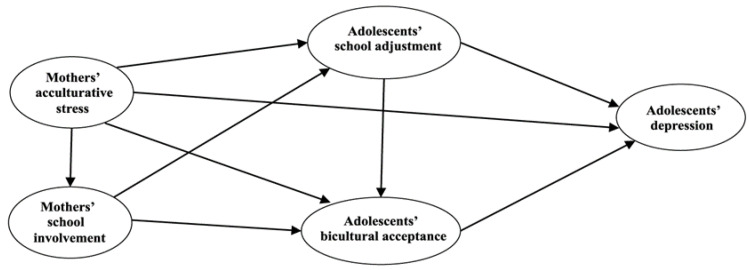
Research model.

Figure 1 illustrates the proposed research model, which depicts the hypothetical relationships among mothers’ acculturative stress as the independent variable; adolescents’ depression as the dependent variable; and mothers’ school involvement, adolescents’ school adjustment, and adolescents’ bicultural acceptance as the mediating variables.

## 2. Materials and Methods

### 2.1. Participants and Procedure

This study used data from the Multicultural Adolescents Panel Study (MAPS) conducted by the National Youth Policy Institute. The MAPS subjects are children of international marriages and their mothers; in particular, most of the children were born in Korea. The MAPS started to collect data from fourth-grade children in Korean elementary schools and their mothers in multicultural families in 2011. Since then, annual follow-ups have been conducted with the participants about their physical and psychosocial characteristics, acculturation factors, parenting, family relationships, and school life. The MAPS data from 2011 until 2019 can be obtained with permission from the National Youth Policy Institute, after submitting a data use request [54]. This study used the data collected in 2014, when the participating children were in the first grade of middle school. Specifically, it included 1238 first-grade middle school students (605 boys, 633 girls; *M* = 12.97, *SD* = 0.35) and their immigrant mothers from multicultural families, who responded to all study variables, as the research participants.

Table 1 presents the participants’ background characteristics. The average age of the participating mothers was 43.52 years (*SD* = 5.13), and the majority were in their 40s (66.4%). As for the mothers’ country of origin, Japan (36.9%) was the most common, followed by the Philippines (25.3%), ethnic Koreans of China (19.1%), and China (6.9%). In addition, regarding the mothers’ final educational attainment, high school graduation (46.8%) was the most prevalent, followed by second- or third-year college graduation or higher (42.2%) and middle school graduation or lower (11.0%). Most of the mothers perceived themselves as having a high level of Korean language proficiency (67.1%). Conversely, most of the adolescents perceived themselves as having a low level of proficiency regarding their foreign mothers’ native language (71.0%), whereas almost all of them considered themselves as having a high level of Korean language proficiency (97.5%). Average monthly household income was KRW 2.45 million (*SD* = 119.29), with KRW 2–3.99 million being the most prevalent (60.9%).

### 2.2. Measures

#### 2.2.1. Depression

Depression perceived by adolescents was measured with the depression scale used in Lee et al.’s [55] study, which is a modified Korean version of Symptom Checklist-90-Revision [56]. The depression scale consists of 10 items, each answered on a four-point scale ranging from not at all (1) to extremely (4). Each of the items comprises questions assessing an individual’s degree of depression (e.g., “I was bothered by feeling no interest in things for the past week”). The possible score range is 10–40, with higher scores indicating a higher level of depression. The internal consistency coefficient (Cronbach’s α) of the depression scale was 0.92.

In this study, three observed variables (three parcels), calculated by the random item parceling method as used by many scholars [57], were used in the structural equation modeling (SEM) analysis for the depression indices; the use of item parceling in SEM analysis helps ensure the normality of data while reducing random errors [57].

#### 2.2.2. Acculturative Stress

The degree of acculturative stress perceived by mothers was measured using the Acculturative Stress Scale developed by Sandhu and Asrabadi [58] and translated into Korean and adapted by Lee [59]. The scale consists of the following three subscales: perceived discrimination (two items; e.g., “I feel that I receive unequal treatment in social situations”), homesickness (three items; e.g., “I miss the people and country of my origin”), and social isolation and inferiority (three items; e.g., “I am made to feel inferior in this society, because I am from a foreign country”). Each item is rated on a five-point scale ranging from strongly disagree (1) to strongly agree (5). The possible score range is 2–10 for perceived discrimination and 3–15 for homesickness as well as social isolation and inferiority. Higher scores indicate greater levels of acculturative stress perceived by mothers. The overall internal consistency coefficient (Cronbach’s α) of the scale was 0.88; the Cronbach’s α was 0.88 for the perceived discrimination subscale, 0.84 for the homesickness subscale, and 0.76 for the social isolation and inferiority subscale. This study used perceived discrimination, homesickness, and social isolation and inferiority as the indices of acculturative stress in the SEM analysis.

#### 2.2.3. School Involvement

The participating mothers’ self-reported degree of school involvement was measured using a scale developed by the MAPS research team. This scale comprises nine items on the levels of school activity participation for supporting school education and operation (e.g., participation in parent meetings and parent education) and communicating with schools (e.g., conversations between parents and teachers about their children). Each item is rated on a four-point scale ranging from never (1) to four or more times a year (4). The possible score range is 9–36; higher scores indicate greater levels of mothers’ participation in school activities and communication with schools and teachers. The internal consistency coefficient (Cronbach’s α) of the scale was 0.87. Moreover, as with the depression scale, the three observed variables, produced by the random item parceling method, were used in the SEM analysis as the indices of school involvement.

#### 2.2.4. School Adjustment

Adolescents’ school adjustment was measured using the scale developed by Kim et al. [60]. This scale is divided into three subscales, each of which consists of five items: learning activities (e.g., “I do not miss school homework”), peer relationships (e.g., “I get along well with my classmates”), and relationships with teachers (e.g., “I am comfortable talking with my teachers”). Each item is answered on a four-point scale ranging from strongly disagree (1) to strongly agree (4). The possible score range of each scale is 5–20, with higher scores indicating higher levels of adjustment in learning activities and relationships with peers and teachers at school. The overall internal consistency coefficient (Cronbach’s α) of the scale was 0.87; additionally, the Cronbach’s α was 0.77 for the learning activities subscale, 0.60 for the peer relationships subscale, and 0.89 for the relationships with teachers subscale. In this study, learning activities, peer relationships, and relationships with teachers were added as the indices of school adjustment into the SEM analysis.

#### 2.2.5. Bicultural Acceptance

The degree of adolescents’ bicultural acceptance was measured using the scale originally developed by Nho and Hong [23] and modified by the MAPS research team. This scale consists of 10 items that measure adolescents’ acceptance of the Korean culture and the culture of their mothers’ countries of origin (e.g., “Learning the Korean culture is important to me” and “It is important for me to learn the culture of my non-Korean parent’s country of origin”). Each item is answered on a four-point scale ranging from strongly disagree (1) to strongly agree (4). The possible score range is 10–40, with higher scores indicating greater levels of bicultural acceptance for Korea and the mother’s country of origin. The internal consistency coefficient (Cronbach’s α) of the scale was 0.74. Additionally, three observed variables, produced by the random item parceling method, were also used in the SEM analysis as the indices of bicultural acceptance.

### 2.3. Statistical Analysis

The data were analyzed using IBM SPSS Statistics (version 26.0) and AMOS 26.0 (IBM Co., Armonk, NY, USA). The participants’ background characteristics and general tendency of the measured variables were analyzed using descriptive statistics, and the Pearson correlation coefficients among the variables were computed. In addition, *t*-tests were conducted to determine if the levels of mothers’ school involvement, adolescents’ school adjustment, adolescents’ bicultural acceptance, and adolescents’ depression differed by the sex of adolescents.

Furthermore, an SEM analysis was performed to examine the direct effect of the participating mothers’ acculturative stress on adolescents’ depression as well as the mediating effects of mothers’ school involvement, adolescents’ school adjustment, and adolescents’ bicultural acceptance on the relationship between mothers’ acculturative stress and adolescents’ depression. Maximum likelihood estimation was used for the SEM analysis, and the model fitness was determined based on the comparative fit index (CFI), Tucker–Lewis index (TLI), and root mean square error of approximation (RMSEA). In general, CFI and TLI values over 0.90 and an RMSEA value below 0.08 indicate a good fit [61]. A bootstrapping procedure was applied to investigate the overall significance level of the indirect effects of mothers’ acculturative stress on adolescents’ depression through mothers’ school involvement, adolescents’ school adjustment, and adolescents’ bicultural acceptance; however, the AMOS bootstrapping procedure does not verify the statistical significance of each potential pathway among mothers’ acculturative stress, mothers’ school involvement, adolescents’ school adjustment, adolescents’ bicultural acceptance, and adolescents’ depression. To overcome this limitation, this study applied the method suggested by Bae [62] that incorporates phantom variables. Phantom variables are used to statistically verify specific indirect effects without influencing the fit of the model and its parameters.

In addition, a multigroup analysis was conducted to investigate whether the structural relationships among mothers’ acculturative stress, mothers’ school involvement, adolescents’ school adjustment, adolescents’ bicultural acceptance, and adolescents’ depression differed by the sex of adolescents. This analysis was performed by sequentially verifying configural, metric, and structural invariances [63]. A configural invariance test was first conducted to examine whether the final research model had a good fit for the groups of male and female adolescents. If configural invariance was supported, the next step was to test metric invariance to find whether the same constructs were being measured across the groups. The metric invariance test was performed by comparing the goodness-of-fit indices (the *χ^2^* values) between the constraint model, in which equality constraints were imposed on all the factor loadings of observed variables, and the baseline model with no equality constraints. After metric invariance was verified, a structural invariance test was conducted to examine whether there were differences between male and female adolescent groups in all the path coefficients indicating the magnitudes of relationships between the latent variables hypothesized in the research model. Specifically, the structural invariance test was conducted by comparing the goodness-of-fit indices (the *χ^2^* values) between the constraint model, in which equality constraints were imposed on all the path coefficients, and the metric invariance model (designated as the baseline model), wherein equality constraints were imposed on all the factor loadings of observed variables.

Finally, mothers’ Korean language proficiency and household income, which have been regarded as variables affecting adolescents’ depression in previous studies [64,65], were also added into the SEM analysis as control variables.

## 3. Results

### 3.1. Descriptive Statistics and Correlations

Table 2 displays the means and standard deviations (*SD*) of the study variables and correlations among them. First, the mean of each of the mothers’ acculturative stress subscales (five-point scale) was low, with perceived discrimination at 2.63 (*SD* = 0.90), homesickness at 2.37 (*SD* = 0.88), and social isolation and inferiority at 2.32 (*SD* = 0.99). The mean of mothers’ school involvement was low at 1.43 (*SD* = 0.42) out of a possible score of four. The mean of each of the adolescents’ school adjustment subscales (four-point scale) was relatively high, with learning activities at 2.88 (*SD* = 0.53), peer relationships at 3.18 (*SD* = 0.41), and relationships with teachers at 3.11 (*SD* = 0.57). The mean of adolescents’ bicultural acceptance (*M* = 2.93, *SD* = 0.39) was above the midpoint of the possible score of four. By contrast, the mean of adolescents’ depression was low at 1.64 (*SD* = 0.54), which was below the midpoint of the possible score of four. Furthermore, while female adolescents had significantly higher levels of bicultural acceptance (*t* = −2.39, *p* < 0.05) and depression (*t* = −3.79, *p* < 0.001) than male adolescents, the levels of the mothers’ school involvement and adolescents’ school adjustment subscales did not differ by the sex of adolescents.

To determine whether the measures of the variables satisfied the assumption of multivariate normality in the SEM analysis, this study examined their skewness and kurtosis. The results showed that the variables passed the threshold for normality suggested by West et al. [66] (the absolute skewness value was below two and the absolute kurtosis value was below seven).

The analysis of the correlations among the variables (see Table 2) performed before the SEM analysis showed that all the subscales of acculturative stress (i.e., perceived discrimination, homesickness, and social isolation and inferiority) were significantly and positively correlated with the depression scale. However, while the homesickness and social isolation and inferiority subscales were both significantly and negatively correlated with the school involvement scale, the perceived discrimination subscale was not. Additionally, the social isolation and inferiority subscale was significantly and negatively correlated with all the subscales of school adjustment (i.e., learning activities, peer relationships, and relationships with teachers), whereas the perceived discrimination and homesickness subscales were negatively correlated with the learning activities and peer relationships subscales. All the subscales of acculturative stress had statistically significant and negative correlations with the bicultural acceptance scale. Moreover, the school involvement scale was significantly and positively correlated with all the subscales of school adjustment and the bicultural acceptance scale. All the subscales of school adjustment had statistically significant and positive correlations with the bicultural acceptance scale, with all the school adjustment subscales being negatively correlated with the depression scale. Furthermore, the bicultural acceptance scale was negatively correlated with the depression scale.

### 3.2. Direct and Indirect Relationships between Mothers’ Acculturative Stress and Adolescents’ Depression: Mediating Roles of Mothers’ School Involvement, Adolescents’ School Adjustment, and Adolescents’ Bicultural Acceptance

An SEM analysis was conducted to examine the pathways from mothers’ acculturative stress, through mothers’ school involvement, adolescents’ school adjustment, and adolescents’ bicultural acceptance, to adolescents’ depression. First, to determine whether the sample data were appropriate for the hypothesized research model, the model’s fit was examined. The results showed a relatively good fit: *χ^2^* = 242.683 (*df* = 80), CFI = 0.977, TLI = 0.970, RMSEA = 0.041. Moreover, the standardized factor loading values of the observed variables ranged from 0.73 to 0.82 for mothers’ acculturative stress, 0.71 to 0.79 for mothers’ school involvement, 0.64 to 0.77 for adolescents’ school adjustment, 0.68 to 0.74 for adolescents’ bicultural acceptance, and 0.81 to 0.86 for adolescents’ depression. Therefore, all the standardized factor loading values of the measured variables were above 0.50, the threshold suggested by Hair et al. [67]. The findings showed that the study data fit the research model.

**Figure 2:**
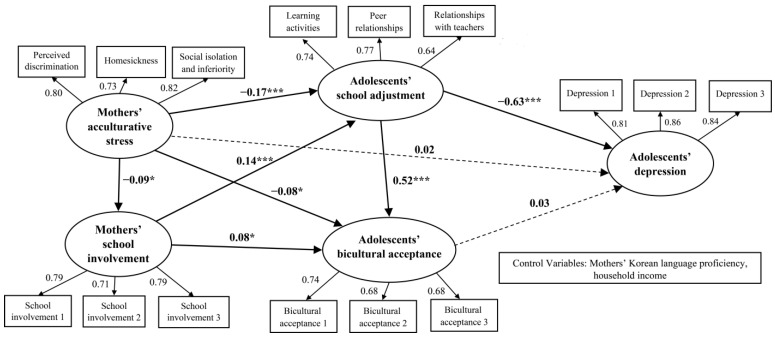
The SEM analysis of the research model (*N* = 1238). * *p* < 0.05; *** *p* < 0.001.

Next, Figure 2 and Table 3 display the results of the SEM analysis for the study model; the fit of the model was fairly robust, *χ^2^* = 316.525 (*df* = 107), CFI = 0.971, TLI = 0.963, RMSEA = 0.040, and seven of the nine paths were found to be statistically significant at *p* < 0.05 and *p* < 0.001. First, mothers’ acculturative stress was significantly and negatively related to their school involvement (*β* = −0.09, *p* < 0.05), and mothers’ school involvement was significantly and positively related to adolescents’ school adjustment (*β* = 0.14, *p* < 0.001) and bicultural acceptance (*β* = 0.08, *p* < 0.05). Moreover, adolescents’ school adjustment had a positive relationship with their bicultural acceptance (*β* = 0.52, *p* < 0.001), but was negatively related to their depression (*β* = −0.63, *p* < 0.001). Therefore, a pathway from mothers’ acculturative stress, through mothers’ school involvement and adolescents’ school adjustment, to adolescents’ depression was identified (mothers’ acculturative stress → mothers’ school involvement → adolescents’ school adjustment → adolescents’ depression).

Second, mothers’ acculturative stress had a significant and negative relationship with adolescents’ school adjustment (*β* = −0.17, *p* < 0.001) and bicultural acceptance (*β* = −0.08, *p* < 0.05). However, mothers’ acculturative stress was not significantly related to adolescents’ depression, with the relationship between adolescents’ bicultural acceptance and depression being insignificant. As such, a pathway from mothers’ acculturative stress through adolescents’ school adjustment to adolescents’ depression was revealed (mothers’ acculturative stress → adolescents’ school adjustment → adolescents’ depression).

To verify the statistical significance of the indirect effects of mothers’ acculturative stress on adolescents’ depression through mothers’ school involvement, adolescents’ school adjustment, and adolescents’ bicultural acceptance, a bootstrapping procedure was applied. The results showed that the overall indirect effect was statistically significant (*b* = 0.08, *p* < 0.01). Next, phantom variables were used to verify the statistical significance of specific indirect effects. The results indicated that two mediated models were statistically significant (Table 4). Specifically, Hypothesis 2 (mothers’ acculturative stress → mothers’ school involvement → adolescents’ school adjustment → adolescents’ depression) and Hypothesis 5 (mothers’ acculturative stress → adolescents’ school adjustment → adolescents’ depression) were supported.

### 3.3. Differences in the Structural Relationships among Mothers’ Acculturative Stress, Mothers’ School Involvement, Adolescents’ School Adjustment, Adolescents’ Bicultural Acceptance, and Adolescents’ Depression according to the Sex of Adolescents

Next, a multigroup analysis was performed to determine whether the structural relationships among mothers’ acculturative stress, mothers’ school involvement, adolescents’ school adjustment, adolescents’ bicultural acceptance, and adolescents’ depression differed by the sex of adolescents. First, the results of the configural invariance test indicated that the final model’s fit was good for the groups of male and female adolescents (males: *χ^2^* = 230.851, *df* = 107, CFI = 0.965, TLI = 0.956, RMSEA = 0.044; females: *χ^2^* = 205.840, *df* = 107, CFI = 0.973, TLI = 0.966, RMSEA = 0.038); thus, configural invariance was verified.

Then, the metric and structural invariance tests were conducted for both groups (Table 5). First, the metric invariance test was conducted to ensure that the same constructs were being measured across these groups. The difference in the *χ^2^* statistics (∆*χ^2^* = 21.981, ∆*df* = 10) between the full metric invariance model, in which equality constraints were imposed on all the factor loading coefficients of observed variables, and the baseline model with no equality constraints was statistically significant. Therefore, full metric invariance was rejected. Next, this study tested the difference in the *χ^2^* statistics between the partial metric invariance model, in which equality constraints were released for only the four variables with the largest differences in factor loading coefficients between the groups of male and female adolescents (i.e., Depression 1, Depression 3, Relationships with teachers, and Bicultural acceptance 1), and the baseline model. When full metric invariance is not supported, it has become common practice to conduct the partial metric invariance test, which accepts some violations of metric invariance (e.g., releasing the constraints on one or more factor loadings) [68]. The difference in the *χ^2^* statistics (∆*χ^2^* =6.902, ∆*df* = 6) was not statistically significant; as such, partial metric invariance was verified. The next step was to test structural invariance to check whether the path coefficients differed according to the sex of adolescents. The difference in the *χ^2^* statistics between the structural invariance model, wherein equality constraints were imposed on all the path coefficients, and the partial metric invariance model, designated as the baseline model, was statistically significant (∆*χ^2^* = 18.597, ∆*df* = 9), which indicated that one or more of the path coefficients between the groups of male and female adolescents differed.

Then, to identify which path coefficient differed by the sex of adolescents, the *χ^2^* difference test between the partial structural invariance model, in which an equality constraint was imposed on each path coefficient, and the partial metric invariance model was sequentially conducted (Table 6). The results showed statistically significant differences in the two path coefficients between the groups of male and female adolescents. Specifically, the negative effect of adolescents’ school adjustment on their depression was greater among girls than it was among boys, whereas the negative effect of adolescents’ bicultural acceptance on their depression was significant only among boys.

## 4. Discussion

The present study examined the mediating effects of mothers’ school involvement, adolescents’ school adjustment, and adolescents’ bicultural acceptance on the relationship between mothers’ acculturative stress and adolescents’ depression, as well as whether the structural relationships differed by the sex of adolescents.

First, mothers’ acculturative stress did not have a direct effect on adolescents’ depression; thus, Hypothesis 1 was not supported. However, this study identified two pathways in which mothers’ acculturative stress exerted indirect influences on adolescents’ depression through mothers’ school involvement and adolescents’ school adjustment. This finding supports the family stress model and family systems theory, both of which show the processes by which the environmental stress experienced by parents affects their children’s psychosocial adjustment through parenting behaviors. In addition, this result is consistent with the findings of several studies that have reported that parental stress (e.g., acculturative and economic stress) affects their children’s behavioral problems indirectly through parenting factors [7,29].

Second, the indirect pathway suggested in Hypothesis 2 (mothers’ acculturative stress → mothers’ school involvement → adolescents’ school adjustment → adolescents’ depression) was supported. This result is in line with earlier research findings showing that immigrant parents’ lower levels of acculturative stress predict higher school involvement [9,37], which subsequently leads to greater school adjustment and academic achievement among their children [9,32,38]. Furthermore, this study’s finding, namely, that adolescents’ greater school adjustment was associated with lower levels of depression, supports the results of several previous studies indicating the negative association between adolescents’ school adjustment and their depression [20,21].

Third, this study also verified Hypothesis 5, which proposed a pathway from mothers’ acculturative stress, through adolescents’ school adjustment, to their depression (mothers’ acculturative stress → adolescents’ school adjustment → adolescents’ depression). This result is also consistent with the findings of earlier studies that have reported that mothers’ higher acculturative stress has a negative influence on school adjustment for children and adolescents [10,44] and that adolescents’ school adjustment is negatively associated with their depression [20,21]. These results can be explained by the negative crossover process of family systems theory [30] and broaden-and-build theory of positive emotions [69]. Specifically, mothers’ high levels of acculturative stress and the resulting depression and negative emotions could be directly transmitted to their children through crossover processes, leading to feelings of helplessness among the children and their school maladjustment. In addition, according to the broaden-and-build theory of positive emotions, the more positive experiences an individual has in daily life, the wider their range of thoughts and behaviors and the more they display prosocial behaviors toward others; however, the more negative experiences they have, the more they focus on their feelings and desires and the more they show an attitude of withdrawal and indifference in interpersonal relationships [69]. Therefore, mothers who experience greater levels of acculturative stress exert negative influences on their children’s psychosocial adjustment by failing to properly respond to their expectations and demands and provide adequate support for their school life.

However, this study’s result indicated that the effect of adolescents’ bicultural acceptance on their depression was not supported. The finding is inconsistent with the results of several prior studies showing that adolescents’ higher bicultural acceptance is associated with lower levels of depression [27,40]. Therefore, Hypotheses 3, 4, 6, and 7, which included a path from bicultural acceptance to depression, were not supported. In general, this study’s results showed that the effect of adolescents’ school adjustment on their depression was stronger than that of their bicultural acceptance among multicultural families. This finding is in line with Yang et al.’s study [70] reporting that peer support has a greater influence on the depression of children from multicultural families than does bicultural acceptance. These results may be explained by the developmental tasks experienced by adolescents from multicultural families and the cultural characteristics of their families. The adolescents from multicultural families participating in this study were first-grade middle school students, who faced developmental tasks to adapt to a school environment and academic works that differed from those at elementary school [12]. In addition, the participating adolescents were the children of multicultural families formed through international marriages between Korean fathers and immigrant mothers; most of the adolescents were born in Korea and were familiar with the Korean culture. Therefore, unlike children from immigrant families who need to quickly adapt to the mainstream culture after their entire family migrates to the host country, school adjustment is the most important developmental task for the participating adolescents, as is the case for their same-age peers from non-multicultural families. As such, the participating adolescents’ school adjustment had a stronger effect on their depression than did their bicultural acceptance.

Fourth, the findings of the multigroup analysis showed that the negative effect of adolescents’ school adjustment on their depression was greater among girls than among boys, whereas the negative effect of adolescents’ bicultural acceptance on their depression was significant only among boys. These results support the research findings of Turjeman et al. [53] indicating that school connectedness and social support have stronger influences on depression among girls than among boys, whereas acculturation difficulties have a stronger effect on depression among boys than among girls. The differences in the effects of adolescents’ school adjustment and bicultural acceptance on their levels of depression by their sex can be explained by the gender socialization process. Girls are traditionally socialized to be emotionally expressive, more empathetic to others’ emotions, and highly oriented to interpersonal relationships and social belongingness [49,53]. This explains why the effects of school adjustment and relationships with peers and teachers on depression are stronger among girls than among boys. By contrast, boys are socialized to be more action-oriented, develop instrumental traits (e.g., assertiveness and independence), and more oriented to achieve personal goals. As a result of this socialization process, male adolescents from immigrant and multicultural families may perceive the process of acculturation as a task to be accomplished [53]. Notably, both parents in multicultural families in Korea show more support of and expectations for social achievement and career exploration for boys than they do for girls, and there still exists a socio-cultural stereotype that primarily imposes the role of family provider onto men [10]. Furthermore, given that when adolescents from immigrant and multicultural families have integrated attitudes toward both the heritage and mainstream cultures, they have higher levels of psychosocial adjustment in the host countries [16,22], boys are more likely than girls to approach biculturalism as a task to be accomplished to meet both parents’ expectations and achieve social and professional success in Korea.

In sum, based on the family stress model and family systems theory, this study contributes to the literature by identifying multiple pathways in which immigrant mothers’ acculturative stress influences adolescents’ depression through the mediators of mothers’ school involvement and adolescents’ school adjustment in multicultural families in Korea. In addition, it is meaningful to verify that the effects of adolescents’ school adjustment and bicultural acceptance on their levels of depression differ by their sex. In particular, unlike many earlier studies that have focused on a single or few individual or environmental variables affecting depression in the children of immigrant and multicultural families, this study comprehensively explores the complex effects of social and cultural factors (i.e., parenting, school, and culture) on depression among adolescents from multicultural families.

The present study’s findings have practical implications for schools and local communities. First, it is necessary to increase the degree of mothers’ participation in school activities and communication with teachers for the adolescents of multicultural families to adjust to school and have good psychological health. However, given that immigrant mothers’ school involvement may weaken their support for children and parenting efficacy due to the territorial behaviors of mothers from non-multicultural families and their prejudices against multicultural families [71], school authorities should support immigrant mothers’ school involvement by considering their backgrounds and characteristics. Specifically, for immigrant mothers to obtain useful information about their children’s school life through school involvement and thus adequately support their children’s school adjustment, school authorities could introduce parents with similar immigrant backgrounds or who are fluent in foreign languages as school support mentors to immigrant mothers. School authorities could also operate school support groups for immigrant parents in connection with local organizations and nearby schools. In addition, many school teachers have been found to be passive when communicating with immigrant parents and have lower expectations of the academic achievement and school adjustment for students from multicultural families than for students from non-multicultural families [72]. As such, multicultural teacher education should be strengthened to reduce prejudice against students from multicultural families and improve the level of multicultural acceptance. Furthermore, local governments should increase academic support for children of multicultural families by using local volunteers, such as university students, in community-based learning programs and expand opportunities for immigrant mothers to work as experts in foreign languages or multicultural education in the community. Especially, as translation services for foreign parents of multicultural families are provided by schools in some regions of Korea [73], it is necessary to expand these services nationwide.

It may also be useful to consider the differential effects of adolescents’ school adjustment and bicultural acceptance on their levels of depression by their sex when running counseling programs for students from multicultural families. For instance, counselors can improve the psychological health of female adolescents from multicultural families by helping them enhance their social networks, such as using school peers and university students with similar cultural backgrounds as peer counselors. For male adolescents from multicultural families, school teachers and counselors could provide activities and tasks that promote a sense of accomplishment and increase bicultural acceptance, such as exploring the cultural values of their mothers’ native countries together and introducing bilingual or intercultural education programs to learn their mothers’ native languages and cultures.

This study offers several future research directions in light of its limitations. First, in this study, measures of school-based involvement and communication between mothers and schools were used as the indices of maternal school involvement. However, several studies have shown that when the form of parental school involvement changes from “hand-on” participation in school activities to support for school life at home (e.g., conversations about school life and support for homework), it has more positive effects on adolescent children’s school adjustment and academic achievement, because their autonomy and independence from parents increase [74,75]. Therefore, future research could examine the effects of both the school- and the home-based involvement of immigrant mothers on their children’s school adjustment and depression. Furthermore, measures of maternal school involvement used in this study were derived from the participating mothers’ self-reported data. However, as parents and children could have different perceptions of identical parenting behaviors [76], future research should investigate the effects of children’s perceived parental involvement on their school adjustment and depression. Third, future prospective studies could provide further evidence of the causal relationships by applying a longitudinal approach to the hypotheses presented in this study to develop an effective intervention program aimed at the psychosocial adjustment of adolescents from multicultural and immigrant families. In addition, this study examined the effects of parenting behaviors (school involvement) of foreign mothers on their adolescent children’s depression. Among the majority of Korean families, mothers were reported to assume primary responsibility for raising children and the time mothers spent raising children was three times higher than that of fathers [77]. However, given that the relationships with fathers are also associated with depression in adolescents from multicultural families [78], more attempts should be made to explore the effects of the parenting behaviors of both parents on their children’s psychosocial adjustment. Lastly, the concept of acculturation this study adopted is based on Berry’s model. Even though Berry’s model is still used as the basic theoretical framework for acculturation studies, its oversimplified and static understanding of dynamic and continuous cultural adaptation that is not solely limited to new migrants has received criticism [79,80]. Rudmin [81] criticized Berry’s model for overlooking the diverse ways of acculturation and formulated sixteen strategies instead of four. In addition, Bhatia [82] argues that it is possible to have the ideas of assimilation, integration, and separation simultaneously inside the self, if political and cultural factors provoke such configuration. Furthermore, Gamsakhurdia’s [80] concept of “proculturation” is noteworthy as an alternative concept to Berry’s acculturation, which is based on the dialogical self and social representation theories from a developmental perspective. Gamsakhurdia [80] argues that previous acculturation theories do not account for how individuals adapt to new cultures through subjective dialogical interaction and how new cultural elements are integrated with social representation. Proculturation has been defined as the continuous process of reconstruction of the self after meeting and dialog with any kind of new cultural elements. This process could entail creation of new social representations or simply reconstruction of older ones [79,80]. Specifically, as proculturation can begin in adolescence when the exploration of self-identity is a major developmental task [79], future research should examine how adolescent children from multicultural or immigrant families acquire their cultural identities through proculturation.

## 5. Conclusions

This study examined the effect of immigrant mothers’ acculturative stress on adolescents’ depression as well as the multiple mediating effects of mothers’ school involvement, adolescents’ school adjustment, and adolescents’ bicultural acceptance using the MAPS data. It also identified whether these structural relationships differed by the sex of adolescents. The findings indicated that mothers’ acculturative stress was indirectly and positively related to adolescents’ depression through both the serial mediations of mothers’ school involvement and adolescents’ school adjustment and the individual mediation of adolescents’ school adjustment. Furthermore, adolescents’ school adjustment had a greater effect on depression among female adolescents than it did among male adolescents, whereas bicultural acceptance had a greater effect on depression among male adolescents than it did among female adolescents. To promote multicultural adolescents’ psychological health and alleviate depression, school authorities and local governments should improve mothers’ school involvement and adolescents’ school adjustment by using school support and mentor groups for immigrant mothers and enhancing multicultural teacher education and school- and community-based academic support for adolescents. In particular, it is necessary to increase the effectiveness of school counseling programs aimed at enhancing the psychosocial health of adolescents from multicultural families by applying intervention strategies that consider the differential effects of their school adjustment and bicultural acceptance on their levels of depression by their sex.

## Figures and Tables

**Table 1:** Background characteristics of participants (*N* = 1238).

	Background Characteristics	Categories	Frequency (%)
Mothers	Age	Under 30 years old	16 (1.2)
		Between 30 and 39 years	250 (20.2)
		Between 40 and 49 years	822 (66.4)
		Between 50 and 59 years	148 (12.0)
		Above 60 years old	2 (0.2)
	Country of origin	Japan	458 (36.9)
		Philippines	313 (25.3)
		Ethnic Koreans of China	236 (19.1)
		China	85 (6.9)
		Thailand	47 (3.8)
		Others (Vietnam, etc.)	99 (8.0)
	Final educational attainment	Middle school graduation or lower	136 (11.0)
		High school graduation	579 (46.8)
		Second- or third-year college graduation or higher	523 (42.2)
	Korean language proficiency	High level	830 (67.1)
		Medium level	315 (25.4)
		Low level	93 (7.5)
Adolescents	Korean language proficiency	High level	1207 (97.5)
		Medium level	19 (1.5)
		Low level	12 (1.0)
	Proficiency in foreign mothers’	High level	210 (17.0)
	native language	Medium level	149 (12.0)
		Low level	879 (71.0)
Household income (KRW)		Under 2 million	349 (28.2)
		Between 2 and 3.99 million	754 (60.9)
		Between 4 and 5.99 million	122 (9.9)
		Above 6 million	13 (1.0)

**Table 2:** Descriptive statistics and correlations among the variables (*N* = 1238).

Variables	1-1.	1-2.	1-3.	2.	3-1.	3-2.	3-3.	4.	5.
1-1. DISC									
1-2. HOM	0.59 **								
1-3. SII	0.66 **	0.59 **							
2. SCHINV	−0.02	−0.10 **	−0.10 **						
3-1. SLE	−0.07 *	−0.15 **	−0.14 **	0.14 **					
3-2. SPE	−0.10 **	−0.14 **	−0.13 **	0.08 **	0.56 **				
3-3. STE	−0.05	−0.03	−0.08 **	0.09 **	0.47 **	0.50 **			
4. BICA	−0.07 *	−0.12 **	−0.10 **	0.10 **	0.34 **	0.35 **	0.32 **		
5. DEP	0.07 *	0.10 **	0.09 **	−0.02	−0.45 **	−0.45 **	−0.35 **	−0.27 **	
*M*	2.63	2.37	2.32	1.43	2.88	3.18	3.11	2.93	1.64
*SD*	0.90	0.88	0.99	0.42	0.53	0.41	0.57	0.39	0.54
Skewness	0.12	0.42	0.49	1.09	−0.08	0.02	−0.25	0.06	0.53
Kurtosis	−0.56	−0.27	−0.37	0.85	0.16	0.02	0.34	0.76	−0.41

1-1. DISC = Perceived discrimination (acculturative stress); 1-2. HOM = Homesickness (acculturative stress); 1-3. SII = Social isolation and inferiority (acculturative stress); 2. SCHINV = School involvement; 3-1. SLE = Learning activities (school adjustment); 3-2. SPE = Peer relationships (school adjustment); 3-3. STE = Relationships with teachers (school adjustment); 4. BICA = Bicultural acceptance; 5. DEP = Depression. * *p* < 0.05; ** *p* < 0.01.

**Table 3:** Maximum likelihood parameter estimates for the SEM analysis (*N* = 1238).

Paths	*b*	*β*	S.E.	C.R.
Mothers’ acculturative stress → Mothers’ school involvement	−0.05	−0.09 *	0.02	−2.58
Mothers’ acculturative stress → Adolescents’ school adjustment	−0.09	−0.17 ***	0.02	−4.82
Mothers’ acculturative stress → Adolescents’ depression	0.01	0.02	0.02	0.78
Mothers’ acculturative stress → Adolescents’ bicultural acceptance	−0.03	−0.08 *	0.01	−2.41
Mothers’ school involvement → Adolescents’ school adjustment	0.15	0.14 ***	0.04	3.97
Mothers’ school involvement → Adolescents’ bicultural acceptance	0.06	0.08 *	0.03	2.21
Adolescents’ school adjustment → Adolescents’ bicultural acceptance	0.40	0.52 ***	0.03	12.20
Adolescents’ school adjustment → Adolescents’ depression	−0.84	−0.63 ***	0.06	−13.67
Adolescents’ bicultural acceptance → Adolescents’ depression	0.04	0.03	0.07	0.62

S.E. = Standard error; C.R. = Critical ratio. * *p* < 0.05; *** *p* < 0.001.

**Table 4:** Statistical significance tests of the indirect pathways (*N* = 1238).

Indirect Pathways	*b*
Mothers’ acculturative stress → Mothers’ school involvement → Adolescents’ school adjustment → Adolescents’ depression	0.01 *
Mothers’ acculturative stress → Adolescents’ school adjustment → Adolescents’ depression	0.07 **

* *p* < 0.05; ** *p* < 0.01.

**Table 5:** Results of the metric and structural invariance tests (*N* = 1238).

Models	*χ^2^* (*df*)	Δ*χ^2^* (Δ*df*)	CFI	TLI	RMSEA
Baseline model	436.692 (214)	-	0.969	0.961	0.029
Full metric invariance model	458.673 (224)	21.981 (10) *	0.967	0.960	0.029
Partial metric invariance model	443.594 (220)	6.902 (6)	0.969	0.962	0.029
Structural invariance model	462.191 (229)	18.597 (9) *	0.968	0.962	0.029

* *p* < 0.05.

**Table 6:** Tests of the differences in the path coefficients by the sex of adolescents (*N* = 1238).

Paths	Boys (*n* = 605)	Girls (*n* = 633)	*χ^2^* (*df*)	Δ*χ^2^* (Δ*df*)
	*b* (*β*)	*b* (*β*)		
Mothers’ acculturative stress → Mothers’ school involvement	−0.04 (−0.08)	−0.04 (−0.09) *	443.595 (221)	0.001 (1)
Mothers’ acculturative stress → Adolescents’ school adjustment	−0.08 (−0.15) **	−0.10 (−0.19) ***	443.740 (221)	0.146 (1)
Mothers’ acculturative stress → Adolescents’ depression	−0.02 (−0.03)	0.04 (0.05)	445.119 (221)	1.525 (1)
Mothers’ acculturative stress → Adolescents’ bicultural acceptance	−0.04 (−0.09) *	−0.05 (−0.10) *	443.809 (221)	0.215 (1)
Mothers’ school involvement → Adolescents’ school adjustment	0.14 (0.14) **	0.15 (0.14) **	443.596 (221)	0.002 (1)
Mothers’ school involvement → Adolescents’ bicultural acceptance	0.02 (0.03)	0.09 (0.12) *	445.431 (221)	1.837 (1)
Adolescents’ school adjustment → Adolescents’ bicultural acceptance	0.44 (0.53) ***	0.37 (0.51) ***	444.320 (221)	0.726 (1)
Adolescents’ school adjustment → Adolescents’ depression	−0.66 (−0.52) ***	−0.99 (−0.71) ***	454.687 (221)	11.093 (1) **
Adolescents’ bicultural acceptance → Adolescents’ depression	−0.18 (−0.12) *	0.04 (0.05)	452.448 (221)	8.854 (1) **

* *p* < 0.05; ** *p* < 0.01; *** *p* < 0.001.

## Data Availability

This research was conducted using publicly available national data (https://www.nypi.re.kr/archive/mps/program/examinDataCode/view?menuId=MENU00226&pageNum=1&titleId=146&schType=0&schText=&firstCategory=&secondCategory=, accessed on 10 February 2023), as mentioned in the Materials and Methods section. The data can be accessed with permission from the National Youth Policy Institute.

## References

[1] Lee H. (2005). Marriage Migration to South Korea: Issues, Problems, and Responses. Korea J. Popul. Stud..

[2] Encyclopedia of Korean Culture. Definition of Multicultural Family. http://encykorea.aks.ac.kr/Contents/Item/E0068878.

[3] Kim B. Current Status of Marriage Immigrants in Korea and Policy Tasks. http://www.nationsworld.kr/bbs/board.php?bo_table=academy&wr_id=55708&sst=wr_hit&sod=desc&sop=and&page=1.

[4] Cho H. (2010). Multiculturalism and Female Marriage Immigrants in Korea. J. Eurasian Stud..

[5] Korean Women’s Development Institute Marriage Immigrants by Nationality in 2021. https://gsis.kwdi.re.kr/statHtml/statHtml.do?orgId=338&tblId=DT_5CA0510N.

[6] Korean Educational Statistics Number of Multicultural Students. https://kess.kedi.re.kr/eng/index.

[7] Bao J., Greder K. (2023). Economic Pressure and Parent Acculturative Stress: Effects on Rural Midwestern Low-Income Latinx Child Behaviors. J. Fam. Econ. Issues.

[8] Hou Y., Kim S.Y., Wang Y. (2016). Parental Acculturative Stressors and Adolescent Adjustment through Interparental and Parent-Child Relationships in Chinese American Families. J. Youth Adolesc..

[9] Lim Y. (2019). Effect of Married Immigrant Women’s Acculturative Stress on School Adjustment in Adolescent Children: Mediating Roles of School Involvement and Parenting Efficacy. Korean J. Hum. Ecol..

[10] Lim Y. (2021). Relationship between Marriage Immigrant Mothers’ Acculturative Stress and their Adolescent Children’s Career Decidedness in South Korea: Mediating Roles of Parenting and School Adjustment. Sustainability.

[11] Park Y.-H., Lee S.D. (2017). Exploring the Predictors of School Adjustment and Academic Achievement during Elementary to Middle-School Transition for Adolescents in Multicultural Families. Korean Educ. Inq..

[12] Yoo S. (2008). Factors Affecting School Adjustment of Middle School Freshmen. Korean J. Youth Stud..

[13] Korea Youth Counseling & Welfare Institute A Year after COVID-19, a Record of Changes in Adolescent Mental Health. https://www.kyci.or.kr/fileup/issuepaper/IssuePaper_202105.pdf.

[14] Kang S. (2022). Adolescent Depression and Anxiety are Getting Worse… How to Regain “Healthy Mentality”?. Chosun Media..

[15] Kim J., Kong B., Kang J., Moon J., Jeon D., Kang E., Ju H., Lee Y., Jung D. (2015). Comparative Study of Adolescents’ Mental Health between Multicultural Family and Monocultural Family in Korea. J. Korean Acad. Child Adolesc. Psychiatry.

[16] Schachner M.K., Van de Vijver F.J.R., Noack P. (2018). Acculturation and School Adjustment of Early-Adolescent Immigrant Boys and Girls in Germany: Conditions in School, Family, and Ethnic Group. J. Early Adolesc..

[17] Park H. (2014). Mediated Effects of Acculturative Stress and Maternal Parenting Stress on Internalized Problems of Children in Multicultural Families: Focused on the Mediating Role of Mothers’ Depression. J. Sch. Soc. Work.

[18] Berry J.W. (2005). Acculturation: Living Successfully in Two Cultures. Int. J. Intercult. Relat..

[19] Schwartz S.J., Unger J.B., Zamboanga B.L., Szapocznik J. (2010). Rethinking the Concept of Acculturation: Implications for Theory and Research. Am. Psychol..

[20] Park J., Lee M. (2014). The Effects of Adolescents’ School Adjustment on Depression in their Transitional Period. J. Fam. Better Life.

[21] So S., Song M., Kim C. (2010). The Effects of Perceived Parenting Behavior and School Adjustment on the Depression of Early Adolescent. Korean J. Youth Stud..

[22] Kwak J.Y. (2020). A Study of School Adaptation among Children of Multicultural Families in the Subtypes of Bicultural Acceptance Attitudes. Korean J. Soc. Welf. Res..

[23] Nho C.R., Hong J.J. (2006). Adaptation of Migrant Workers’ Children to Korean Society: About Adaptation of Mongolian Migrant Worker’s Children in Seoul, Gyeong gi Area. J. Korean Soc. Child Welf..

[24] Han S., Yang M., Park Y. (2020). Exploring the Longitudinal Changes of Multicultural Adolescents’ Achievement Motivation by the Developmental Patterns of their Bicultural Acceptance. J. Educ. Cult..

[25] Kang L., Choi N., Kang S. (2021). Minority Language Proficiency of Multicultural Adolescents: The Effects of Bicultural Acceptance Attitudes, Parents’ Educational Support, and the Use of the Minority Language at Home. Fam. Environ. Res..

[26] Lee H.-H., Yun J.-M., Han J.-Y. (2021). Verification of the Longitudinal Relationship between Mothers’ Cultural Adaptation Patterns, Multicultural Acceptability of Multicultural Adolescents, and National Identity: Focusing on the Mediating Effect of the Autoregressive Cross-Lagged Model. J. Digit. Converg..

[27] Yu H.-Y., Paik J. (2022). The Effect of Bicultural Acceptance Attitude on Depression of Multicultural Adolescent: Mediating Effect of Social Withdrawal. J. Ind. Converg..

[28] Conger R.D., Elder G.H.J. (1994). Families in Troubled Times: Adapting to Change in Rural America.

[29] Lorenzo-Blanco E.I., Meca A., Unger J.B., Romero A., Gonzales-Backen M., Piña-Watson B., Cano M.Á., Zamboanga B.L., Des Rosiers S.E., Soto D.W. (2016). Latino Parent Acculturation Stress: Longitudinal Effects on Family Functioning and Youth Emotional and Behavioral Health. J. Fam. Psychol..

[30] Bakker A.B., Demerouti E., Grzywacz J.G., Demerouti E. (2013). The Spillover-Crossover Model. New Frontiers in Work and Family Research.

[31] Salo V.C., Schunck S.J., Humphreys K.L. (2020). Depressive Symptoms in Parents are Associated with Reduced Empathy toward Their Young Children. PLoS ONE.

[32] Kim S., Lee S., Koo Y., Lee K. (2016). The Effect of Parental School Involvement on Children’s School Adjustment: The Mediating Role of Parental Efficacy. Korean J. Hum. Dev..

[33] Hoover-Dempsey K.V., Sandler H.M. (1995). Parental Involvement in Children’s Education: Why does It Make a Difference?. Teach. Coll. Rec..

[34] Hill N.E., Taylor L.C. (2004). Parental School Involvement and Children’s Academic Achievement: Pragmatics and Issues. Curr. Dir. Psychol. Sci..

[35] Lee W.-S. (2019). The Effects of Multicultural Families Parents Participation in School Activities to Children’s School Adaptation. Multicult. Diaspora Stud..

[36] Ku H., Mo K.-H., Seong S.-H. (2020). A Study on How the School Activity Participation of Parents with Migrant Background can Affect their Parenting Efficacy. J. Educ. Cult..

[37] Youn B., Kim K.-K. (2012). Determinants of International Marriage Migrant Women’s Participation in School Activities at the Elementary School Level. Korean J. Sociol. Educ..

[38] Cheung C.S. (2019). Parents’ Involvement and Adolescents’ School Adjustment: Teacher-Student Relationships as a Mechanism of Change. Sch. Psychol..

[39] Rutland A., Cameron L., Jugert P., Nigbur D., Brown R., Watters C., Hossain R., Landau A., Le Touze D. (2012). Group Identity and Peer Relations: A Longitudinal Study of Group Identity, Perceived Peer Acceptance, and Friendships amongst Ethnic Minority English Children. Br. J. Dev. Psychol..

[40] Tikhonov A.A., Espinosa A., Huynh Q.L., Anglin D.M. (2019). Bicultural Identity Harmony and American Identity are Associated with Positive Mental Health in U.S. Racial and Ethnic Minority Immigrants. Cultur. Divers. Ethnic Minor. Psychol..

[41] Birman D., Weinstein T., Chan W., Beehler S. (2007). Immigrant Youth in US Schools: Opportunities for Prevention. Prev. Res..

[42] Yang Y., Cho S.-S., Lee S.-Y. (2022). The Relationship between Parental Monitoring and Support and Multicultural Acceptability Attitude among Multiethnic Adolescents: Focusing on the Self-Esteem. Multicult. Educ. Stud..

[43] Silinskas G., Kiuru N., Aunola K., Metsäpelto R.-L., Lerkkanen M.-K., Nurmi J.-E. (2020). Maternal Affection Moderates the Associations between Parenting Stress and Early Adolescents’ Externalizing and Internalizing Behavior. J. Early Adolesc..

[44] Han S., Xue Y., Cheng C., Park Y. (2017). Relationships among Immigrant Women’s Perceived Social Support, Acculturation Stress, and their Children’s School Adjustment. Korean J Educ. Methodol. Stud..

[45] Wang-Schweig M., Miller B.A. (2021). Examining the Interdependence of Parent-Adolescent Acculturation Gaps on Acculturation-Based Conflict: Using the Actor-Partner Interdependence Model. J. Youth Adolesc..

[46] Kandel D.B., Andrews K. (1987). Processes of Adolescent Socialization by Parents and Peers. Int. J. Addict..

[47] Radke-Yarrow M., Kochanska G., Stein N.L., Leventhal B., Trabasso T. (1990). Anger in Young Children. Psychological and Biological Approaches to Emotion.

[48] Güngör D., Bornstein M.H., Chuang S.S., Tamis-LeMonda C.S. (2013). Gender and Developmental Pathways of Acculturation and Adaptation in Immigrant Adolescents. Gender Roles in Immigrant Families.

[49] Cyranowski J.M., Frank E., Young E., Shear M.K. (2000). Adolescent Onset of the Gender Difference in Lifetime Rates of Major Depression: A Theoretical Model. Arch. Gen. Psychiatry.

[50] Connelly J.P., O’Connell M. (2022). Gender Differences in Vulnerability to Maternal Depression during Early Adolescence: Girls Appear More Susceptible than Boys. Psychol. Sch..

[51] Thompson M.P., Kingree J.B., Desai S. (2004). Gender Differences in Long-Term Health Consequences of Physical Abuse of Children: Data from a Nationally Representative Survey. Am. J. Public Health.

[52] Pizeta F.A., Loureiro S.R., Pasian S.R. (2018). Maternal Depression, Social Vulnerability and Gender: Prediction of Emotional Problems among Schoolchildren. J. Child Fam. Stud..

[53] Turjeman H., Mesch G., Fishman G. (2008). The Effect of Acculturation on Depressive Moods: Immigrant Boys and Girls during their Transition from Late Adolescence to Early Adulthood. Int. J. Psychol..

[54] NYPI Youth and Children Data Archive. Multicultural Adolescents Panel Study. https://www.nypi.re.kr/archive/mps/program/examinDataCode/view?menuId=MENU00226&pageNum=1&titleId=146&schType=0&schText=&firstCategory=&secondCategory=.

[55] Lee K., Baek H.-J., Lee J., Kim J. (2010). The Annual Report of Korean Children and Youth Panel Survey 2010 (Research Report 11-R10).

[56] Kim G., Kim J., Won H. (1984). Korean Manual of Symptom Checklist-90-Reversion.

[57] Bandalos D.L., Finney S.J., Marcoulides G.A., Schumacker R.E. (2001). Item Parceling Issues in Structural Equation Modeling. New Developments and Techniques in Structural Equation Modeling.

[58] Sandhu D.S., Asrabadi B.R. (1994). Development of an Acculturative Stress Scale for International Students: Preliminary Findings. Psychol. Rep..

[59] Lee S.R. (1997). A Study on Acculturative Stress among North Korean Defectors. Master’s Thesis.

[60] Kim J.K., Baek H.-J., Lim H.-J., Lee K.-O. (2010). Korea Children and Youth Panel Survey 2010 (Research Report 10-R01).

[61] Yu J.P. (2012). Concepts and Knowledge of Structural Equation Modeling.

[62] Bae B.R. (2011). Structural Equation Modeling with Amos 19: Principles and Practice.

[63] Kline R.B. (2011). Principles and Practices of Structural Equation Modeling.

[64] Kim K.-H. (2019). Effect of Family Income on Academic Achievement, Depression Anxiety, and Aggression Focusing on Panel Comparison of Children and Adolescents. J. Sch. Soc. Work.

[65] Kim Y. (2021). The Effect of Parents’ Korean Proficiency on their Children’s Psychological Health within Multicultural Families: Focusing on the Mediated Effect of the Communication Level between Parents and their Children. Master’s Thesis.

[66] West S.G., Finch J.F., Curran P.J., Hoyle R.H. (1995). Structural Equation Models with Nonnormal Variables: Problems and Remedies. Structural Equation Modeling: Concepts, Issues, and Applications.

[67] Hair J., Black W., Babin B., Anderson R., Tatham R. (2006). Multivariate Data Analysis.

[68] Putnick D.L., Bornstein M.H. (2016). Measurement Invariance Conventions and Reporting: The State of the Art and Future Directions for Psychological Research. Dev. Rev..

[69] Fredrickson B.L. (2001). The Role of Positive Emotions in Positive Psychology. The Broaden-and-Build Theory of Positive Emotions. Am. Psychol..

[70] Yang S.-Y., Park S.-K., Kim M.-S. (2013). Effects of Bicultural Characteristics and Social Capital on Psychological Adaptation. J. Korea Contents Assoc..

[71] Lee A.M., Rhee C.-W. (2017). Community Participation Affects Marriage Immigrant Women’s Parenting Efficacy. Korean J. Fam. Soc. Work.

[72] Baik M.-W., Ha M.-J. (2016). A Study on Teachers’ Two-Fold Recognition of Students from Multicultural Family. Multicult. Educ. Stud..

[73] Kim D. (2023). Translation Service for Parents of Multicultural Families, Fully Expanded in Elementary, Middle and High Schools. Jejukyeongje Ilbo. http://www.jejukyeongje.com/news/articleView.html?idxno=30416.

[74] Gonida E.N., Cortina K.S. (2014). Parental Involvement in Homework: Relations with Parent and Student Achievement-Related Motivational Beliefs and Achievement. Br. J. Educ. Psychol..

[75] Grover K.A. (2015). A Validation of the Family Involvement Questionnaire-High School Version. Ph.D. Thesis.

[76] Cho J., Ha J.H., Jue J. (2020). Influences of the Differences Between Mothers’ and Children’s Perceptions of Parenting Styles. Front. Psychol..

[77] Lee J. (2022). Awareness of Gender Equality has Improved, But… The Problem of “Women-Centered Parenting” Still Persists. The Segye Times. http://www.segye.com/newsView/20220419510145?OutUrl=naver.

[78] Oh S.-H. (2016). The Study on the Eco-Systemic Variables Affecting Multicultural Family Adolescents’ Depression. J. Community Welf..

[79] Ogoro M., Minescu A., Moriarty M. (2022). Cultural Identity in Bicultural Young Adults in Ireland: A Social Representation Theory Approach. Soc. Sci..

[80] Gamsakhurdia V. (2018). Adaptation in a Dialogical Perspective—From Acculturation to Proculturation. Cult. Psychol..

[81] Rudmin F. (2009). Constructs, Measurements and Models of Acculturation and Acculturative Stress. Int. J. Intercult. Relat..

[82] Bhatia S., Ram A. (2009). Theorizing Identity in Transnational and Diaspora Cultures: A Critical Approach to Acculturation. Int. J. Intercult. Relat..

